# Exploring the intersection of hermeneutics and implementation: a scoping review

**DOI:** 10.1186/s13643-023-02176-7

**Published:** 2023-03-02

**Authors:** Martha L. P. MacLeod, Graham McCaffrey, Erin Wilson, Lela V. Zimmer, David Snadden, Peter Zimmer, Steinunn Jónatansdóttir, Trina M. Fyfe, Erica Koopmans, Cathy Ulrich, Ian D. Graham

**Affiliations:** 1 School of Nursing, University of Northern British Columbia, Prince George, British Columbia Canada; 2 Health Research Institute, University of Northern British Columbia, Prince George, British Columbia Canada; 3 Faculty of Nursing, University of Calgary, Calgary, Alberta Canada; 4 Department of Family Practice, Faculty of Medicine, University of British Columbia, Prince George, British Columbia Canada; 5 Northern Medical Program, Division of Medical Sciences, University of Northern British Columbia, Prince George, British Columbia Canada; 6 University of Northern British Columbia, Prince George, British Columbia Canada; 7 School of Health Sciences, University of Northern British Columbia, Prince George, British Columbia Canada; 8 Division of Medical Sciences, University of Northern British Columbia, Prince George, British Columbia Canada; 9 Geoffrey R. Weller Library, University of Northern British Columbia, Prince George, British Columbia Canada; 10 Northern Health Authority, Prince George, British Columbia Canada; 11 School of Epidemiology and Public Health, University of Ottawa, Ottawa, Ontario Canada; 12 Clinical Epidemiology Program, Ottawa Hospital Research Institute, Ottawa, Ontario Canada

**Keywords:** Hermeneutics, Implementation, Implementing, Scoping review, Health

## Abstract

**Background:**

An enduring challenge remains about how to effectively implement programs, services, or practices. Too often, implementation does not achieve its intended effectiveness, fidelity, and sustainability, even when frameworks or theories determine implementation strategies and actions. A different approach is needed. This scoping review joined two markedly different bodies of literature: implementation and hermeneutics. Implementation is usually depicted as focused, direct, and somewhat linear, while hermeneutics attends to the messiness of everyday experience and human interaction. Both, however, are concerned with practical solutions to real-life problems. The purpose of the scoping review was to summarize existing knowledge on how a hermeneutic approach has informed the process of implementing health programs, services, or practices.

**Methods:**

We completed a scoping review by taking a Gadamerian hermeneutic approach to the JBI scoping review method. Following a pilot search, we searched eight health-related electronic databases using broadly stated terms such as implementation and hermeneutics. A diverse research team that included a patient and healthcare leader, working in pairs, independently screened titles/abstracts and full-text articles. Through the use of inclusion criteria and full-team dialogue, we selected the final articles and identified their characteristics, hermeneutic features, and implementation components.

**Results:**

Electronic searches resulted in 2871 unique studies. After full-text screening, we retained six articles that addressed both hermeneutics and implementing a program, service, or practice. The studies varied widely in location, topic, implementation strategies, and hermeneutic approach. All addressed assumptions underpinning implementation, the human dimensions of implementing, power differentials, and knowledge creation during implementation. All studies addressed issues foundational to implementing such as cross-cultural communication and surfacing and addressing tensions during processes of change. The studies showed how creating conceptual knowledge was a precursor to concrete, instrumental knowledge for action and behavioral change. Finally, each study demonstrated how the hermeneutic process of the fusion of horizons created new understandings needed for implementation.

**Conclusions:**

Hermeneutics and implementation have rarely been combined. The studies reveal important features that can contribute to implementation success. Implementers and implementation research may benefit from understanding, articulating, and communicating hermeneutic approaches that foster the relational and contextual foundations necessary for successful implementation.

**Trial registration:**

The protocol was registered at the Centre for Open Science on September 10, 2019.

MacLeod M, Snadden D, McCaffrey G, Zimmer L, Wilson E, Graham I, et al. A hermeneutic approach to advancing implementation science: a scoping review protocol 2019. Accessed at osf.io/eac37.

**Supplementary Information:**

The online version contains supplementary material available at 10.1186/s13643-023-02176-7.

## Background

In 2016, the British Columbia (BC) Patient-Oriented Research (POR) Support for People and Patient Oriented Research and Trials (SUPPORT) Unit created the Knowledge Translation-Implementation Science (KT-IS) Methods Cluster to advance patient-oriented KT-IS methodological approaches [1, 2]. At the same time, research team members, including a patient, with theoretical and practical expertise in philosophical hermeneutics, clinical practice, and/or implementation had noticed the importance of specific, contextualized human interaction and relationships when implementing programs, services, or practices. We had found that careful attention to the micropolitics of influence within specific contexts [3, 4] contributed to successful implementation in ways that went well beyond the standardization and tailoring of experimental and quasi-experimental designs preferred in implementation science [5, 6]. We noted that a hermeneutic approach may directly attend to the inherent complexity and contextuality of implementation but hermeneutic studies have seldom directly addressed questions of implementation.

Our scoping review was initiated to explore the intersection of hermeneutics and implementation in the context of health. The intent was to understand the characteristics of a body of literature [7] about implementing health programs, services, or practices that used some form of hermeneutics in study design and through this the review, gain new insights about implementation.

### Hermeneutics

Hermeneutics, applied to health research, is an interpretive approach that foregrounds dialogue, temporality, and context in understanding how people make sense of their particular situation. Hermeneutics means interpretation. In its modern form, it is a philosophy of how human beings arrive at understandings of the world and our experience of it, based on constantly interpreting what our contexts, senses, thoughts, and feelings tell us [8–10]. It holds that how we interpret our experiences is always influenced by what has formed our outlook in the past, both personally and culturally. For example, healthcare professionals of different disciplines tend to emphasize different values and ways of identifying and solving problems.

A second fundamental assumption in hermeneutics is that language deeply influences our interpretations of the world. The words we use, even to ourselves in forming thoughts, express what we mean and at the same time entail choices about words we do not use. (Do I say “hypertension” or “high blood pressure” to a patient, and what might my choice convey to the other person?). Gadamer [8, 9], the most prominent philosopher of hermeneutics, put conversation at the center of his account of how we can arrive at new understandings. If we enter into dialogue with someone else about a topic, with enough open-mindedness to listen to what the other person has to say, then we might come away from the conversation thinking differently. Gadamer used a metaphor of “horizons” to convey this principle. If I change position, or step to a higher spot, then my horizon, what I can see, shifts. The importance of conversation is one aspect of hermeneutics that makes it useful for health professionals and healthcare settings. Much of what health professionals do, including when implementing evidence into practice, requires well-conducted and well-timed, clinically relevant conversations that include the patient’s values, context, concerns, and priorities and may require a shift from one’s original position or agenda.

Hermeneutic research, which draws on hermeneutic philosophy for its methods, can prompt the articulation of taken-for-granted practices of how learning occurs, how evidence is taken up, how knowledge evolves, and how actions are taken or not taken over time in particular places and situations [11, 12]. A hermeneutic approach has the potential to help us understand the human face of implementation, especially as what can seem straightforward to implement, can be more complicated than anticipated.

### Implementation

Implementation can be understood to be “actively planned and deliberately initiated effort[s] with the intention to bring a given object into practice” (p. 110) [13]. Those who lead implementation usually structure these efforts through specific strategies within a particular context. Implementation success is generally evident in “adoption, uptake, or sustainability” [13]. We consider the object brought into practice to be evidence, knowledge that has been tested and found to be credible and derived from sources that encompass research, providers’ experience, patient experience, and local information [14].

At this time, most studies of implementation are concerned with “determinants, processes and effects of implementation interventions” [15], frequently focusing on changes in behavior [5]. Accordingly, experimental or quasi-experimental designs that can demonstrate the effectiveness of particular implementation interventions are prevalent. Qualitative designs that are commonly used in implementation research “examine different aspects of interventions and context which contribute to effectiveness” [16] and are expected to have strong links to, or refinement of, existing theory or conceptual frameworks. Other qualitative approaches are created to inform the design, prediction, or evaluation of complex interventions, and often extend or test theory [17]. Despite the increasing inclusion of systems thinking and complexity theory in implementation, a linear approach to learning and behavioral change during the uptake of evidence in practice prevails [18]. Linearity is frequently at odds with the knowledge to be gained, the way people learn, and the complexity of implementing change within organizations [19, 20].

Despite their importance, everyday interactions at the clinical interface, where evidence is taken up in complex situations, may be minimally addressed or overlooked in implementation studies [21]. Attention to the process of implementation is often focused on components of the implementation that influence outcomes [22] or facilitators and barriers to implementation [23]. Process evaluations, sometimes considered to be “sibling” studies of implementation studies [22], are designed to capture these aspects. Process evaluations usually attend to reach, dose delivered/received, fidelity, co-intervention, contamination, and additional contextual factors [22, 24, 25]. Studies are increasingly identifying relevant attributes of context [26], but the interplay of context and implementation success has received limited attention [19, 20, 27].

With few exceptions [28] researchers have identified the discrete components of implementation rather than paying direct attention to how implementing a program, service, or practice actually happens over time. For example, few have explored the pacing of implementing. Instead, studies have focused on time as a discrete entity, such as total time of contact, a collection of data at time points [29], time as a resource measured in minutes or hours [25], or total time taken for an intervention [25]. Conversations between implementers and practitioners that may take place at the clinical interface during implementation are seldom detailed in the literature [25, 28]. More recently, however, researchers who partner with patients [30] or who use collaborative inquiry approaches to bring knowledge into practice [20, 28] have addressed processes of implementation in their studies.

### Objectives

We conducted a broad, systematic scoping review with an a priori search protocol as the concepts of hermeneutics and implementation had not been explored together before. Although most hermeneutic research is qualitative in nature, it could not be assumed a priori that evidence about hermeneutics and implementation would be exclusively qualitative.

The objectives were to:

- Systematically explore the extent of literature that uses a hermeneutic approach to implementing in the context of health
- Map the publications by country of origin, approach to hermeneutics and key features in hermeneutic approaches to implementing
- Inform the development of approaches to implementing programs, services, or practices

## Methods

The methods were informed by the JBI approach [7, 31], which builds on Arksey and O’Malley [32] and Levac et al. [33], and by the hermeneutic approach of Boell and Cecez-Kecmanovic [34, 35]. We noted that scoping review methods are increasingly being clarified and refined [7, 36], with recent attention to the involvement of patients in reviews [37].

The team consisted of a patient [PZ] with extensive life experience including implementing programs; academic researchers from several disciplines [IG, MM, GM, DS, EW, LZ], with expertise in hermeneutics and/or implementation; a healthcare leader responsible for health programs and services [CU]; a health research librarian [TF]; and research trainees [SJ, EK]. Unlike many scoping reviews, where research trainees implement the review protocol, all members of the research team, including the patient, were fully involved in all aspects of the review.

An a priori scoping review protocol was registered with the Centre for Open Science (osf.io/eac37). Deviations from the protocol are detailed in the sections below.

Due to a commitment to the hermeneutic approach of remaining open to new possibilities [8, 9, 12, 34], coupled with the unexplored area of what could count as a hermeneutic approach, we used flexible and iterative processes [7, 38] in (1) formulating the research question; (2) screening and identifying relevant literature; (3) selecting relevant studies; (4) charting data into tables; and (5) synthesizing, summarizing, and reporting results.

### Formulating the research question

The formulation of the research question provided a good example of how the research team worked together throughout the review. When the initial search results were inconclusive, the whole team, including the patient, trainees, librarian, and academic researchers, all with differing experiences and disciplines, met to determine the next steps. The discussion was inclusive and iterative, and revolved around each team member articulating their perspectives. The team took time to develop a shared common understanding. That is, the team had a hermeneutic conversation, characterized by listening, dialogue, and coming to new shared horizons. The patient, in this conversation and throughout, asked for clarification and raised the question, what does this mean? He helped others in the group to be confident in expressing what they knew and did not know. As a service user without insider knowledge of the healthcare system, the patient kept bringing often highly conceptual discussions back to concrete realities, asking about implications for those who experience implementation at the clinical interface. There were many such extensive discussions over the course of the review.

The question was formulated around PCC: Population or Participants, Concept and Context [7, 31]. The team kept the question broad to remain open to potential links between hermeneutics and implementation. In a preliminary search of research databases and the grey literature, the librarian found no research literature that explicitly linked the two concepts. Although hermeneutics was mentioned in the health literature, the term, hermeneutics, was not found in the implementation literature.

The original question identified in the protocol was, “How is philosophical hermeneutics currently taken up in implementation science or the context of implementing?” We changed the term to “hermeneutics” because the term “philosophical hermeneutics” is often associated with the work of a specific philosopher [8].

We therefore identified the population or participants as any participants; the concepts as hermeneutics and implementing; and the context as health programs, services, or practices. The research question became,

What constitutes a hermeneutic approach to the process of implementing health programs, services or practices?

### Screening and identifying relevant literature

#### Inclusion/exclusion criteria

In keeping with a hermeneutic approach [8, 9, 34, 35], precise a priori inclusion and exclusion criteria were not identified. We proceeded with concepts that were sensitizing rather than operational, to avoid pre-determining or limiting the inclusion of articles that could deepen or extend our understanding of the nexus of hermeneutics and implementation.

Through multiple conversations among team members which drew on disciplinary knowledge, other literature, and experience, we sharpened inclusion criteria in the process of selecting articles. We sought to balance feasibility, breadth, and comprehensiveness. In reviewing articles, we always asked, what meaning does this particular article bring in relation to our understanding of the intersection of implementation and hermeneutics?

Detailed documentation and frequent communication among the whole team assisted in maintaining consistent interpretation of the criteria.

##### Population or participants

All populations or participants concerned with health were included, with anticipation that primary populations or participants would be patients, healthcare providers, communities, and decision makers.

##### Concept

Each publication had to include both hermeneutics and a component of implementing or implementation.

**Hermeneutics**

- For an article to be considered hermeneutic it needed an explicit theoretical statement of being based in hermeneutics. It also had to explicitly address context, temporality, dialogue, and personal understanding. That is, there was an indication of where implementation was happening, who was involved and in what ways they were involved; there was a mention of change over time; there was an indication of conversation or dialogue among individuals intended to foster understanding; and it was evident that there was a change in participants’ understanding. The articles needed to state that interpretation was used and reflect interpretation in how the article was written. It needed to be evident that links had been made beyond the immediate situation to new understandings of theory, or a new articulation of experience or to gaining a new point of view. Articles needed to state interpretation and links to the philosophical hermeneutic underpinnings, as well as demonstrate these aspects. The way in which hermeneutics was expressed in the article was used as an inclusion criteria. If an article was not “sufficiently hermeneutic” it was dropped from consideration [39].

**Implementing**

- As the goal was to study the actual process of implementing, that is adaption, adoption, improvement, decision-making, or communication that affects practices and/or behaviors [40, 41], articles needed to include such features as what the authors/researchers did, how they changed practice, etc. Articles needed to report more than just a change in attitude, but also could include decision-making, a change in behavior, or descriptions of applying action, plan, or recommendations for change. The studies did not need to be primary studies of implementing.

##### Context

We did not focus on a particular concept of health, but noted that implementation could occur within health programs, health services, or practices, in any setting, and with any population or participants. We included all health settings, including community, acute care, long-term care, primary care, as well as some settings less frequently associated with health, such as education of health professionals. No exclusions were based on geographical or locational factors, cultural factors, specific race, gender, or sex-based interests.

### Article types, study designs, and language

Primary studies and reviews were eligible. Included were academic journal papers and brief reports from the health sector. Conference abstracts, editorials, opinion pieces, commentaries, and philosophical or theoretical papers were excluded. Grey literature was also excluded following the preliminary search that resulted in no relevant documents. Included was literature published in five languages: English, Icelandic, Norwegian, Swedish, and Danish, because all research team members were fluent in English and one was fluent in the other four languages.

### Identifying the literature

As a result of the iterative process, the librarian performed a simple search of the literature using subject headings, if available, for hermeneutics and a search for the term hermeneutic in the title (ti) and/or abstract (ab). We then situated hermeneutics within a search of simple implementation language in the title and/or abstract: implement* or adapt* or adopt* or improve*. Examples of the search are provided in Table 1.

**Table 1:** Literature search strategy

MEDLINE OVID	
1. exp hermeneutics/ (310)	
2. hermenutic*.ti,ab. (3,585)	
3. 1 or 2. (3307)	
4. (implement* or adapt* or adopt* or improve*).ti,ab. (2,932,305)	
5. 3 and 4. (616)	
Embase OVID	
1. exp hermeneutics/ (320)	
2. hermenutic*.ti,ab. (3,634)	
3. 1 or 2 (3671)	
4. (implement* or adapt* or adopt* or improve*).ti,ab. (3,865,590)	
5. 3 and 4. (703)	
EBM Reviews	
1. hermeneutic*.ti,ab.(18)	
2. (implement* or adapt* or adopt* or improve*).ti,ab. (289,601)	
3. 1 and 2 (9)	
PubMed	
1. Hermeneutic [Title/Abstract] OR "Hermeneutics"[Mesh]) AND (implement[Title/Abstract] OR adapt[Title/Abstract] OR adopt[Title/Abstract] OR (improve[Title/Abstract]) (287)	
CINAHL Ebsco	
1. TI hermeneutic* OR AB hermeneutic*(3,502)	
2. TI ( (implement* or adapt* or adopt* or improve*) ) OR AB ( (implement* or adapt* or adopt* or improve*) ) (663,590)	
3. 1 and 2 (649)	
PsycInfo Ebsco	
1. TI hermeneutic* OR AB hermeneutic*(6,578)	
2. DE "Hermeneutics"(2,048)	
3. 1 OR 2(6,796)	
4. TI ( (implement* or adapt* or adopt* or improve*) ) OR AB ( (implement* or adapt* or adopt* or improve*) ) (695,097)	
5. 3 AND 4 (890)	
Web of Science	
1. TS=hermeneutic* Indexes=SCI-EXPANDED, SSCI, A&HCI, CPCI-S, CPCI-SSH, ESCI Timespan=All years (16,447)	
2. TS= (implement* OR adapt* OR adopt* OR improve*) (6,627,276)	
3. 1 AND 2 (1,301)	
Joanna Briggs Institute (JBI)	
1. hermeneutic*.ti,ab.(0)	
2. hermeneutic*.mp. [mp=text, heading word, subject area node, title](75)	
3. (implement* or adapt* or adopt* or improve*).mp. [mp=text, heading word, subject area node, title] (4,762)	
4. 2 AND 3 (71)	

We conducted the search across eight health-related databases: MEDLINE OVID, Embase OVID, EBM Reviews, PubMed, CINAHL EBSCO, PsycInfo EBSCO, Web of Science, and JBI EBP Database. The search included published literature from conception of databases to June 11, 2021. The original search was completed on February 27, 2019 and updated searches following the same process with the same databases were completed on April 17, 2020 and June 11, 2021. We did not pursue a formal peer review of the search [42] due to the simple search strategy, the team’s expertise in the subject area, and the team’s iterative and thoughtful discussion regarding the hermeneutic and implementation language included in the search.

### Selecting relevant publications

In order to include a broad range of literature, titles and abstracts were reviewed at the same time. Refining or sharpening the inclusion decisions happened through the full involvement of the whole team in all stages of review and through the extensive discussion of what was meant by hermeneutics and implementing. Once articles were identified, titles and abstracts were uploaded into Evidence Partner’s DistillerSR systematic review software for duplicate removal, article screening, selection, and data extraction. Members of the research team: a patient, academic researchers, the healthcare leader, and a research trainee, were split into five pairs to first screen titles and abstracts of articles, and second, to screen the full texts of articles.

### Pilot screening

Pairs of reviewers completed pilot screening with 1% of the articles identified in the search. Reviewers independently considered the titles and abstracts for the concepts of *hermeneutics* and *implementing* within a health context*.* Each pair came to consensus. Reviewers provided a text response for their primary reason for inclusion, exclusion, or uncertainty (“cannot tell”). Following whole team discussion, the text responses were grouped into reasons for inclusion or exclusion and categorized. In addition to type of article, lack of abstract, and language, decisions to exclude occurred in this order: (1) the title or abstract did not concern a health context; (2) hermeneutics was not mentioned; (3) a process of implementing was not identified. As some articles about decision-making had implications for implementation, decision-making was created as a criterion for inclusion.

### Title and abstract screening

The same pairs individually screened titles and abstracts to determine whether articles should proceed to full text review using the same process and priorities as in the pilot screening. Discussions among the whole team clarified questions and confirmed reasons to include articles. Reasons for inclusion were refined. Within the concept of implementing, articles were included that were either about implementing a practice, service, or program; could inform implementation; or were about making decisions relevant to implementing. Rationales for excluding the articles were that they were “not implementation” or “not sufficiently implementing”. Reviewers could select “other” as a reason to include or exclude and provide free text comments. An individual reviewer who was uncertain about inclusion could mark the article as “cannot tell”. Any discrepancies, where one reviewer indicated “include,” resulted in full text screening.

### Full-text screening

Three steps were taken for full text screening. At the first step, each pair individually and independently reviewed each article and decided to include or exclude an article. The primary reasons for inclusion and exclusion at this stage remained the same as for title and abstract screening. Following this review, two other reviewers independently categorized comments in the free text “other” category into existing categories. The whole team reviewed and reached full consensus, agreeing to this categorization, with no additional categories needed.

During the regularly scheduled full research team discussions, as each person contributed their perspective and insights, it became apparent that reviewers picked up different features in the articles and that greater consistency of review was needed as well as equivalent attention paid to hermeneutics and implementing. During those discussions, each person contributed what they found to be indicative of hermeneutics and implementing. Through this open dialogue, with the goal of hearing each other and understanding differences and similarities in perspectives and meaning, the team gained increasing clarity and came to consensus about the features of hermeneutics that needed to be present for the articles to be included for further examination [39].

Following the first full-text review step, experts in hermeneutics and implementation reviewed the articles in a second full-text review step. MM reviewed all included full text articles to confirm the presence of hermeneutics and LZ reviewed the full text articles that MM had reviewed at an earlier stage. If hermeneutics was present, IG reviewed to confirm the presence of implementing.

In the third full text screening step, the whole team made final decisions about inclusion of the remaining articles during a two-day meeting. Research team members read these articles in depth and discussed them in detail for the presence of both hermeneutics and implementing. Through dialogue we reached consensus and gained increased understanding and insights [35] about the nexus between hermeneutics and implementing across included studies.

Consistent with scoping review methods, we did not assess methodological quality [31] or risk of bias [7].

These repeated in-depth discussions led the research team to further articulate what needed to be present to be named as a hermeneutic approach. Articles were not sufficient for inclusion when there was the following: results were presented as descriptions of experiences and/or through simply naming and illustrating themes [43]; the presentation, stated philosophy, or philosophical references were not linked to the approach or findings; hermeneutics was talked about but not reflected in the article. The team also clarified what needed to be in place about implementing. The processes of implementing were present when authors depicted how they actually went through the steps or processes of implementing an intervention or decision-making. We did not consider implementing to be present when the article only depicted the following: participants’ experience of a service, an evaluation of a service, the result of an implementation, or the need for future implementation.

### Charting the data

Three persons charted data from the final remaining articles. The study characteristics as outlined in the protocol were charted by a research trainee (SJ) and confirmed by the research team (Table 2). Two research team members with expertise in hermeneutics (MM, LZ) extracted details on hermeneutic approach and implementation. The categories evolved during discussion of the results, and the whole team refined the charted data accordingly in Tables 3 and 4.

**Table 2:** Characteristics of included studies

Author, year, country	Aim	Setting	Research design/methods	Participants	Hermeneutic approach	Hermeneutics in the study	Focus of implementation
Darbyshire 1994 [44] Scotland	To describe the creation and first year implementation of a nursing course, and hermeneutic evaluation of students’ experiences and learning	BA (Honors) Health Studies Degree Year 4 option course at Glasgow Caledonian University, ‘Understanding Caring Through Arts and Humanities’	Evaluation: Two, 1-h focus group interviews (6–7 persons in each) at the end of the course. Transcribed and interpreted hermeneutically by author	15 students in course and author (teacher in the course)	Heideggerian hermeneutics (Heidegger, Benner, Diekelmann)	Hermeneutic interpretation focusing on how the participants experienced expectations of the course, their experiences of participating and learning, their reactions to the course content and assessment, and how the course might influence or impact on their personal and professional lives	A nursing course
Greenhalgh and Shaw 2017 [45] England	To inform policy by making sense of a complex literature on heart failure and its remote management	No specific geographic or clinical context	Hermeneutic systematic review Interpretive approach to (1) accessing and interpreting the literature; 2) developing an argument	Included were 7 systematic reviews of systematic reviews, 32 systematic reviews (including 17 meta-analyses and 8 qualitative reviews); six mega-trials and over 60 additional relevant empirical studies and commentaries	Boell and Cecez-Kecmanovic’s (2014) [35] approach (derived from Gadamer) for a hermeneutic systematic review	Followed the question, “Is this paper likely to add meaning to our emerging overview of the field?”	Care for persons with heart failure via telehealth
Hughes et al. 2020 [46] England	To inform policy and practice on integrated care by deepening understanding of what integrated care is, how it is experienced, and how it is conceptualized	High income countries	Hermeneutic systematic review using repeated cycles of searching, filtering, and interpretation	Those with multiple chronic conditions associated with aging Included were 31 primary research publications, 22 evidence reviews, 14 theoretical and conceptual reviews, and 4 policy documents	Boell and Cecez-Kecmanovic’s (2010; 2014) [34, 35] approach for a hermeneutic systematic review	Lines of argument of each paper were identified and interpreted across papers using “story lines of research” to make sense of findings	Integrated care or “programs of change” in healthcare systems and organizations that focus on integrating care that facilitate person-centered, relationship-based services
Larsson and Blomqvist 2015 [47] Sweden	To investigate changes over time in an interdisciplinary group that engaged in development work regarding pain and pain assessment	Rehabilitation ward specialising in orthopedics, rheumatology, and multi trauma care	Participatory action research 7 focus group sessions over 5 months Transcribed material analyzed by authors	3 RNs, 2 assistant nurses, 1 physiotherapist	Nyström; Gadamer; Meleis’ transition theory	Hermeneutic analysis: identified meaning units; made tentative interpretation; created main interpretation	A plan for treating patients in pain, including a pain assessment form
Thirsk et al. 2014 [48] Canada	To explore how registered nurses address psychosocial issues for patients and their families living with chronic kidney disease, specifically how nurses’ attributions or explanations, of patient/family behavior influence their subsequent psychosocial intervention	Inpatient nephology unit	Unstructured interviews (to allow for genuine conversations). Questions centred on nurses’ practice and experience of providing psychosocial interventions with patients and families Audio recorded and transcribed verbatim	7 registered nurses	Gadamer; Attribution theory	Hermeneutic inquiry and analysis: Interviewing until patterns became apparent. Reading/re-reading transcripts, interpretive memos, attention to the individual case, discussion of beginning interpretations of patterns; expanding interpretation through literature	Psychosocial interventions with patients and families living with chronic kidney disease
Xiao et al. 2018 [49] Australia	To critically examine how staff and residents initiated effective cross-cultural communication and social cohesion that enabled positive changes to occur	Four aged care homes	Part of a larger critical action research project on developing multicultural workforce in residential care Semi-structured Interviews with residents /families Focus groups with staff over 5 months	23 residents and 7 family members (10 culturally and linguistically diverse; 20 not culturally and linguistically diverse) 56 staff (16 culturally and linguistically diverse; 40 not culturally and linguistically diverse)	Critical hermeneutic analysis Giddens (1984) [50] Structuration Theory Double hermeneutic	Data analysis and interpretation informed by Giddens’ critical concepts and double hermeneutic methodology. First, a generic procedure of thematic analysis through coding, grouping codes, summarising codes into categories and identifying themes. Categories then subjected to a critical reflection on the interplays between structural power and human agencies	Actions and social conditions that could improve cross cultural communication and address the negative consequences of poor cross cultural communication

**Table 3:** Hermeneutic features of included studies

Article	Darbyshire (1994) [44]	Greenhalgh and Shaw (2017) [45]	Hughes et al. (2020) [46]	Larsson and Blomqvist (2015) [47]	Thirsk et al. (2014) [48]	Xiao et. al (2018) [49]
Why a hermeneutic approach is proposed	Developing an interpretive nursing humanities course	Making sense of diverse literature	Making sense of heterogeneous literature, with terms used to mean different things	Examining assumptions in clinical practice	Examining assumptions in clinical practice	Examining cross cultural communication practices
Dialogue	Discursive space of teaching and learning	Dialogue between different kinds of evidence	Dialogue to make sense of different perspectives and contexts and to bring forth challenges of harmonizing conceptual models and practical application strategies	Group process over time using dialogue to articulate and shift clinician understandings of pain	Dialogue as research tool—participant interviews, literature, theory	Dialogue as topic of study and research tool
Expanding understanding	Participants found a new way to understand their experiences of nursing through an exploration of art and literature	“Multivocal”, multi-paradigm approach to reviewing the evidence	A more holistic understanding of a complex phenomenon that is inseparable from context	Participants arrived at better understanding of patient and colleague perspectives	Nurses improved their understanding of patients' lives and contexts	Granular understanding of individualized communication strategies
Context	Post graduate nursing education in Scotland—a course exploring connections between art, literature and the practice of nursing	A broad exploration of the use of telehealth in heart failure care	A broad exploration of integrated care strategies and concepts especially in high income countries, with aging populations.	Acute care (in-patient) rehabilitation unit in Sweden, relieving patient’s pain was identified as a major challenge	Acute care nephrology units in Canada	Long-term care homes in Australia with cultural diversity among residents and staff
Temporality	Time for reflective learning	Living with chronic disease	Growing need for greater alignment of health and social care	Series of group discussions	Living with chronic disease	Development and maintenance of therapeutic relationships
Interpretation	Use of interpretive discussion in teaching; interpretation of course evaluations	Five tensions identified through interpretation of diverse sources in relation to each other	Four main perspectives identified through interpretation of story lines across papers	Participants re-interpreted assumptions through group process	Interpretation of interview data using framework of attribution theory	Interpretation of practitioner behaviour in institutional context using Giddens’ structuration theory
Outcomes	Positive evaluations of reflective, discursive classroom	Highlighted significance of patients’ actual situation in relation to using evidence	Highlighted limitations of conceptual models, the complexity of integrated care and its inseparability from context	Dialogue and reflection were the primary methods used to initiate change	Insight into nurses’ negative perceptions of patients enabled improved patient-centred care	Consideration of context, including social structures and norms

**Table 4:** Implementation components of included studies

Article	Darbyshire (1994) [44]	Greenhalgh and Shaw (2017) [45]	Hughes et al. (2020) [46]	Larsson and Blomqvist (2015) [47]	Thirsk et al. (2014) [48]	Xiao et. al (2018) [49]
Impetus for implementation	To redress the traditional student-teacher power imbalance and to help students and teacher re-consider and re-vision nursing	To make sense of the field of the use of telehealth technology in heart failure management	To make sense of disparate literature on what integrated care is, how it is experienced and how it is conceptualized	To improve care for patients with regard to pain and pain assessment	To understand nurses’ clinical reasoning about psychosocial interventions in order to improve the implementation of family interventions	To address cross cultural communication between residents and staff, one of the most challenging aspects of care in aged care homes
Implementation actions	Students and teacher agreed on mutual expectations and principles through approach taken in weekly discussions of novels, poetry, art, music, paintings, photographs. Specifically focused discussions and essays	Searched the literature using hermeneutic approach. Extracted key data, arguments and explanations. Crafted a narrative synthesis of relevant key questions, theories, methods, findings and scholarly arguments. Surfaced tensions in the literature; identified unanswered questions about implementing telehealth for heart failure	Conducted a hermeneutic literature review. Extracted “story lines” from each of the included papers, including the key questions, theories, arguments, and methods. Identified four perspectives, identified how integration must be understood as emerging from particular as well as common contexts	During focus group sessions, participants made a plan for treating patients in pain, made a decision about a pain assessment form. In the last two sessions participants evaluated the pain assessment form and further developed their work related to pain. All sessions started with feedback from actions from the previous session and participants decided together on way forward	No specific implementation actions	No specific implementation actions
Components influencing implementation	Mutually developed expectations and principles	Hermeneutic approach to searching literature and synthesizing key questions, theories, methods, findings and scholarly arguments	Hermeneutic approach to searching and synthesizing literature on patient experience, concepts, strategies and contextual factors, and lines of argument	Participants’ joint action, reflection, and decision-making about the content and process of the change to be implemented	Nurses’ clinical reasoning processes	Organizational and structural conditions for change
Implementation steps	Specifically focused discussions, activities, and assignments	Informed implementation steps	Informed implementation steps	Working together, and collectively reflecting, participants planned, implemented and evaluated clinical actions and decisions	Informed implementation steps	Informed implementation steps
What are the main findings about implementation that were illuminated by the hermeneutic approach?	The hermeneutic approach fostered interpretive and critical understandings and new insights into previously taken-for-granted issues	The hermeneutic approach to the literature review allowed five tensions in the literature to be surfaced: heart failure as isolated condition vs one within multi-morbidities; intensive monitoring telehealth vs basic support; active self-managing patient of theory vs actual patients of experiential descriptions; biomedically focused practice vs patient experience practice; fixed vs agile telehealth programs. The tensions were key to explaining variation in uptake and sustainability of telehealth services	The hermeneutic approach to the literature review revealed tensions and gaps, including misalignment of professional and organizational efforts with patient experiences/expectations; mis-match between conceptual models and strategies to integrate care. Integrated care is also subject to political economy of health care and power relations. Integrated care can be better understood as an emergent set of practices shaped by the context than as an intervention that will achieve a pre-determined set of outcomes.	The hermeneutic approach worked to transfer knowledge into action. Participation and reflection improved professionals’ understanding of patients’ experiences, and changed attitudes and clinical actions, while creating a tool for further action. Participants changed their understanding of their own professional identity and the group’s confidence and relationships changed in a positive manner	The hermeneutic approach of attending to context and to the patients story helped nurses to overcome attribution bias and improve clinical reasoning.	The critical hermeneutics approach enhanced understanding about social conditions. It illuminated cross cultural care and effective communication. It identified a key concept for addressing issues of structural power imbalance, cultural humility.
Implementation knowledge/outcome	Conceptual knowledge Professional reorientation	Conceptual knowledge Instrumental knowledge (hermeneutic approach to literature review) Implementation knowledge	Conceptual knowledge Instrumental knowledge (hermeneutic approach to literature review) Implementation knowledge	Conceptual knowledge Instrumental knowledge Professional reorientation	Conceptual knowledge Instrumental knowledge for subsequent practice Professional reorientation	Conceptual knowledge Knowledge for subsequent implementation

Legend: Conceptual knowledge is knowledge created for general enlightenment, that indirectly influences actions; instrumental knowledge is knowledge that can be directly applied in specific ways [51, 52]

### Synthesizing, summarizing, and reporting results

Through the extended discussions held in person and subsequently in three multi-hour teleconferences, team members engaged in a hermeneutic interpretation of the results, achieved a common horizon, and answered the research question. The discussions were reflexive and free-flowing, where we shared and questioned our pre-judgements [8, 9], assumptions, and interpretations. Team members used the detailed notes that were taken of all team meetings and teleconferences to reflect on their own and others’ insights and revise and/or deepen their own and our collective understanding. Although stated in the registered protocol, we did not use NVivo to manage data.

## Results

**Fig. 1:**
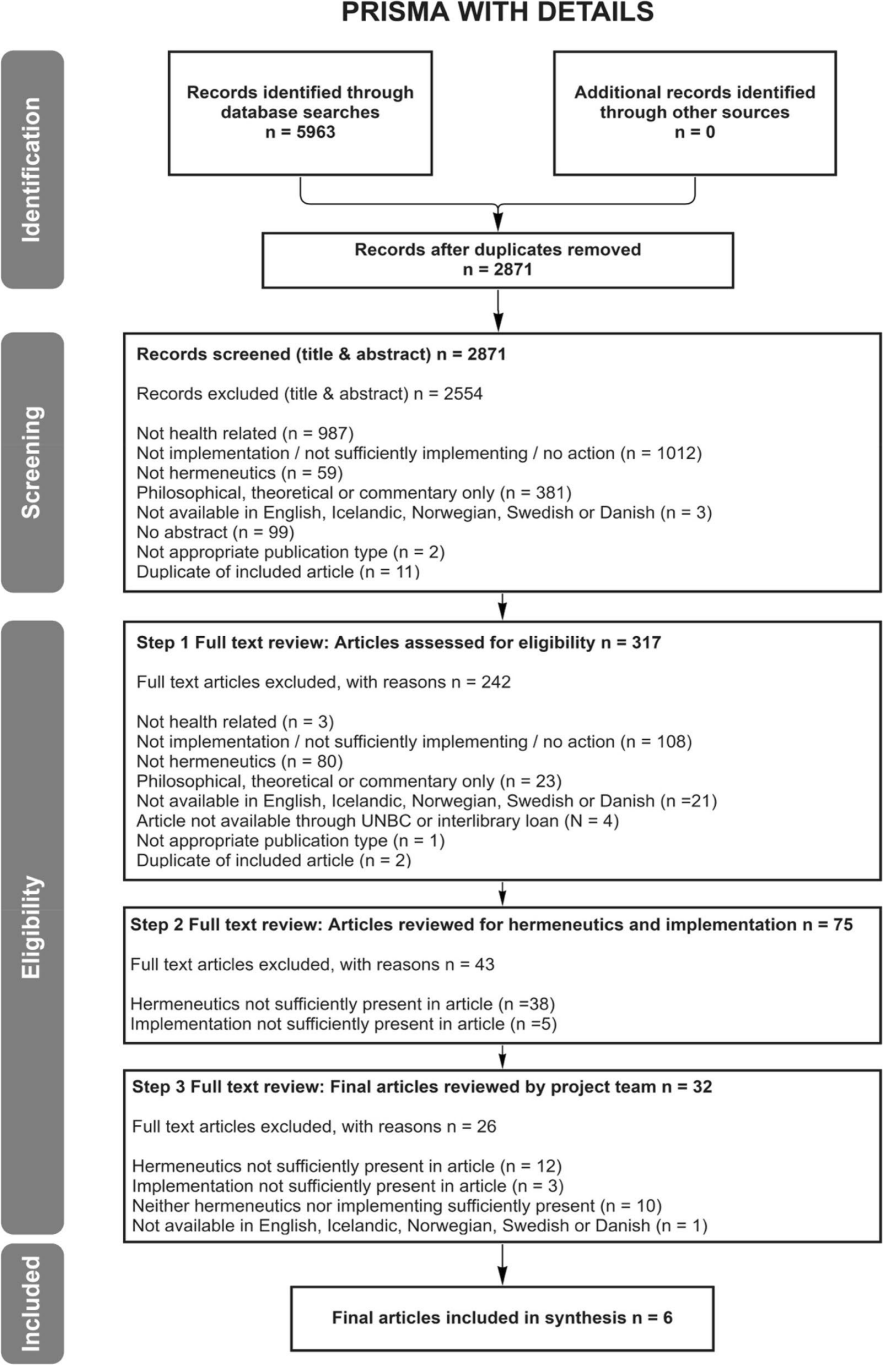
Process of article selection

The search within the eight health-related databases yielded 5963 articles of which 3092 were duplicates, leaving 2871 unique articles. Titles and abstracts of the 2871 articles were reviewed and 2554 excluded. At the first full-text screening stage, 317 articles were assessed for eligibility; 242 articles were excluded. At the second full-text screening stage, 75 articles were specifically reviewed for the presence of hermeneutics and implementation. Few of the articles included all of the features of hermeneutics or were clearly about implementing, nevertheless 32 of the 75 were retained for further discussion by the whole research team. In the third full-text stage, these 32 articles were reviewed by the whole research team at the in-person meeting. Six articles, four single research studies [44, 47–49] and two review studies [45, 46], met the selection criteria. Although the two review studies were completed by the same team, the topics and included articles differed (Fig. 1).

The six included articles were heterogeneous. One study was completed in 1994 [44], with five published between 2014 and 2020. The studies were undertaken in Australia, Canada, England, Scotland, and Sweden, with diverse participants in varied settings. The focus of implementation differed widely among the studies: implementing a nursing course, implementing pain assessment and treatment in a rehabilitation ward, decision-making by RNs in implementing psychosocial kidney care, improving cross cultural communication in aged care homes, caring for persons with heart failure via telehealth, and integrating care in a variety of settings. Participants were nursing students; registered nurses; rehabilitation ward staff; and aged care staff, residents, patients, and families. The two systematic literature reviews [45, 46] did not delineate specific participants, but one [46] identified studies that included patients’ perspectives.

As evident in Table 2, the approach to hermeneutics varied among the studies; each drew on a different set of theorists, including Benner, Diekelmann, Boell and Cecez-Kecmanovic, Meleis, Nyström, and Giddens. The researchers drew on philosophers, namely Gadamer [44, 47, 48] and Heidegger [44]. Xiao et al. [49] took a critical hermeneutic stance [50]. Hermeneutics informed the approach in all of the studies and was particularly evident in two studies’ data analysis and interpretation [47, 49], and throughout all of the steps in four studies [44–46, 48].

### A hermeneutic approach

Hermeneutics was expressed differently in each of the articles albeit with commonalities among them (Table 3). All of the studies identified a hermeneutic approach as an ideal way to address and question a dominant narrative, existing assumptions and practices, either at the systems or institutional level. The single studies sought to explore existing assumptions in clinical practice [47, 48]; create a different way to prepare nurses [44]; and examine organizational assumptions and practices that shape cross-cultural communications [49]. The reviews used a systematic hermeneutic approach to make sense of diverse, substantive bodies of literature [45, 46].

#### Dialogue

Authors used dialogue, listening, conversation, and sharing to develop understanding and implement change, to identify where shifts might be possible, and to make those shifts. All studies used dialogue as a research tool. They used dialogue in planning and implementing the intervention [44, 47]; conversing between different kinds of evidence [45, 48, 49] or framing a phenomenon [46].

#### Context

All advocated for change that considers the “individual case” within a specific context [53]. In each of the studies, organizational contexts [46–49], practice contexts [45, 47–49], or professional contexts [44, 45, 48] influenced implementation and its interpretation.

#### Temporality

All of the authors addressed timing and pacing through which people experienced their engagement in change [54]. The approaches ranged from taking time to listen, question, and change understanding [44], to addressing how patients living with chronic illness could best be served over time [45], to how changes in action or care happened over time [46, 47], to how time played out in the lives and everyday contexts of patients [48], to how time contributed to changing relationships between patients and residents [49].

#### Processes of change

All of the studies described change in fine-grained detail. For example, Greenhalgh and Shaw [45] and Hughes et al. [46] illuminated details of change in the studies they reviewed through their hermeneutic systematic review process. Xiao et al. [49] identified specific steps taken by care aides in Australian care homes to facilitate cross-cultural communication. Although each of the six studies attended to concrete details of life and change in very specific contexts, the authors interpreted these observations in ways that leveraged new insights.

#### Interpretation

The approaches to interpretation differed, from a critical interpretation of literature [45, 46], to interpretation of participants’ words and actions during implementation [44, 47, 49] or decision-making [47]. Interpretation within the implementation process often occurred through a process of dialogue and reflection. Within the studies, authors paid explicit attention to interpretation within the data analysis process [44, 45, 48, 49] as well as within the discussion [44–49]. The interpretive process was sometimes accomplished through placing findings in relation to theory or diverse sources, or in addressing tensions in the literature [45, 46]. Interpretations included identifying and naming key aspects of practice that informed how change could occur [46, 48, 49]. All authors interpreted their study results in light of a theoretical framework or the theoretical or philosophical underpinnings of a particular hermeneutic approach. In each of the studies, this interpretation created an expansion of understanding about possibilities for implementation actions and outcomes that would not otherwise be available.

### Implementation

All six studies concerned implementing evidence into practice and accomplishing that process in different ways. The impetus for implementation in each case was a problematic situation that required new ways of understanding in order to create new approaches to systems of care [46], nursing professional practice [44], clinical reasoning [48], or clinical practice [45, 47, 49]. The studies focused on the organization, practice, or personal components that influenced implementation and its steps (Table 4).

For example, Darbyshire [44] identified that a mutual approach to setting and achieving expectations and principles of conduct could address the imbalanced power structure inherent in professional practice courses. Greenhalgh and Shaw [45] and Hughes et al. [46] sought to make sense of highly disparate fields of literature through a hermeneutic approach that informed changes in clinical practice and expanded the limitations of conventional reviews.

The six studies expressed implementation steps differently. None followed an *a priori* theory or framework. Instead, they informed implementation through specific attention to the context, process, complexity, and reflexivity that was required for implementation [55, 56]. Two studies described implementation steps taken: specifically focused discussions, activities, and assignments [44]; and a series of actions of assessing, planning, implementing, and evaluating, with reflection at each step [47]. The other four studies focused on the initial phase of understanding the context and evidence in ways that would specifically inform the next actions. Greenhalgh and Shaw [45] identified tensions in the literature and specific approaches for further implementation of telehealth; Hughes et al. [46] identified strategies and concepts to inform the integration of care; Thirsk et al. [48] articulated influences on nurses’ clinical reasoning to be incorporated in nurses’ practice; Xiao et al. [49] created understandings that directly informed implementation of the next phase of a project to improve cross cultural communication.

All of the studies created conceptual knowledge, that is new ways of understanding the context and other conditions necessary for implementation. For example, Greenhalgh and Shaw [45] surfaced five tensions in the literature that were key to explaining variation in uptake and sustainability of telehealth service. The tensions were about the condition itself; approaches to monitoring and support; approaches to practice; and types of programs. Importantly, the tension between actual patient experience and idealized patient-as-self-sufficient-agent was identified. By bringing forward essential tensions about these topics, Greenhalgh and Shaw [45] identified the need for effective and sustained implementation efforts that could close otherwise obscured gaps.

In Larsson et al.’s study [47], researchers used participation and reflection to mobilize knowledge into action and establish change. Through prolonged engagement and focus group sessions, participants changed their understanding of the patient and their situation, from an object (e.g., a fracture, a knee), to a whole person. A pain assessment form served as a tool to improve understanding of patients’ experiences of pain. Participants changed their attitudes about their own professional identity, and the group’s confidence and relationships improved. Thirsk et al. [48] showed that attention to context and to the patients’ story helped overcome attribution bias and improved clinical reasoning. Through participation in Darbyshire’s educative process [44], students created a community of learning, developed interpretive and critical understandings, highlighted ethical and professional concerns, and generated new insights into previously taken-for-granted issues. Xiao et al.’s [49] use of critical hermeneutics allowed for enhanced understanding among residents and staff about social conditions that enabled cross cultural communications. It illuminated how the changes implemented through the co-creation of resources by residents, family, and staff could facilitate better cross-cultural care built on effective communication. It showed how cultural humility assisted in addressing issues of structural power imbalance.

In all of the studies, participants and readers of the subsequent publications were invited to view the process of implementing and implementation differently, in a new light.

## Discussion

The six studies that reflected a hermeneutic approach to the process of implementing health programs, services, or practices were highly varied in their location, subject, and approach to hermeneutics. In the included papers, a hermeneutic approach to implementing was based on a set of assumptions not normally at play in implementation science: a focus on essential problems underlying implementation; involving actions over time that are based in relationships, dialogue, and a reflection-action dynamic; creating conceptual knowledge and new ways of understanding that may be necessary for behavior to change over time and evidence to be successfully implemented.

### Assumptions at play

Within conventional approaches to implementation, there remains the underlying assumption that credible evidence is created outside a situation and then implemented, often within complex contexts or environments [22, 55–57]. Such approaches acknowledge the need to identify rigorous implementation processes and strategies, complete with addressing facilitators and barriers to implementation [24, 55–57]. Despite these acknowledgements [18, 22, 57], researchers rarely question the assumptions underpinning conventional implementation approaches and note, but seldom illuminate the messiness of how implementing actually happens over time.

A hermeneutic approach directly addresses the human dimensions of implementing. The underlying assumptions of a hermeneutic approach hold that researchers or implementers and participants are in a reciprocal relationship, in which, through dialogue, conditions are created to enhance or draw out new ways of thinking about issues or problems [9, 12, 39]. There is negotiation through respectful conversation, conducted with humility and an openness to learning. As part of this process of dialogue, a hermeneutic approach to implementing prompts participants to consider the perspective of others, to be self-aware, and to challenge existing assumptions and structures.

With few exceptions, the discussion of power in implementation literature is often absent [23] or discussed only in relation to explanatory or statistical power [57]. Attention to power differentials features in participatory action research, which is increasingly seen in knowledge translation and implementation research [58, 59]. A hermeneutic approach to implementing describes the social positioning of people involved in implementation and at times explicitly addresses power differentials, for example, within patient-professional interactions, among participants, or between research approaches. As well, some hermeneutic approaches to implementing explicitly consider power in relation to the structural influences [50] on the processes and practices of implementing.

### Addressing the big issues: preparing the ground for implementing

Many theories, models, and frameworks include pre-implementation planning, a pre-implementation phase, or assessment of organizational readiness [56, 60, 61]. They usefully detail determinants, specific strategies, required skill sets, contextual factors, and considerations for behavior change, etc. They identify actions to take and importantly, focus largely on *what* to do. A hermeneutic approach, however, focuses on the *how*; that is how to focus one’s attention, how to interact, and how to work with people in a particular context so they speak in their own terms about what matters to them, and in this way, develop a common understanding about possible ways to successfully implement a program, service or practice.

We suggest that a hermeneutic, processual approach could serve to prepare the ground for implementation in two ways. The first is to help surface large, nebulous issues that implementation of a service or practice cannot readily address. The second is to provide a foundational step for planned change, which can happen after the problem is identified and before implementation strategies are thoroughly explored.

A hermeneutic approach to implementing may address amorphous issues, such as re-envisioning an approach to professional education, addressing discrimination, improving cross-cultural communication, altering decision-making processes, or looking at large-scale shifts that are needed in an area of chronic disease care or care integration. Through a hermeneutic approach, those leading the implementation process and those actually implementing the program, service, or practice, raise their knowledge, perceptions, and assumptions and in that way, together prepare the ground for implementation. Through dialogue and listening, they can create common goals with a clearly articulated purpose and approach to change that holds integrity for each implementation situation. These aspects of implementation are often lacking [3, 5, 24]. It is difficult for education, practice, or policy changes to solve problems when underlying assumptions and tensions are not addressed, in terms of what might best serve the persons involved in that time and situation.

Arriving at an intersubjective understanding through dialogue—an intersubjective exchange—has the potential to lead to a common understanding of the problem, appreciation of the issue, common understanding of the evidence or innovation and what the innovation can or cannot do, and recognition of the need for and approach to change. Such common understandings are a precondition for collective action—they prepare the ground for implementation. Through dialogue and acting together in authentic engagement, participants can notice power differences and work them through. Participants can come to a fusion of horizons, a new or common understanding of the problem or issue, and a workable approach to implementation that suits the people and their context.

It is an aphorism that the solution to a problem lies within the problem itself. A hermeneutic approach to implementation attends to the world as it is, rather than the world as imagined. Those making changes can take foundational steps through respectful, inclusive dialogue among those who may think differently; recognizing that common understandings change over time; and that change always builds on the past as it moves into the future. For example, toolkits are increasingly popular as a menu or suite of tools designed to spread innovations [62]. A hermeneutic approach to implementing can, through addressing difference, uncover assumptions and help groups come to decisions about who chooses from a toolbox, what tools to choose, and how to decide what selections to make.

### Implementing through creating knowledge

The six included articles highlight the polyvalent understanding of implementing and a recognition that knowledge is created in practice. A hermeneutic approach, with the focus on an increased awareness of contextual considerations, better knowledge of the other and their perspectives or experiences, contributes most strongly to the conceptual use of knowledge [51, 52]. At the same time, knowledge is developed in its use. The articles all show ways in which conceptual knowledge development can contribute to later instrumental knowledge use, for example, in a more enlightened application of attribution theory in clinical reasoning or being attuned to cultural humility in the provision of organizational supports for cross-cultural communication.

The hermeneutic approach may help participants to gain new experiences, to see a particular situation differently and through reflection inherent in the research and/or reading process, to link evidence or the proposed innovation ‘object’ with their own experience and/or knowledge. With insights developed through reflection and action, new possibilities arise in terms of participants understanding their own context-embedded experience, along with understanding how knowledge may be developed and implemented. In all of the studies, participants were attuned to their experience, and through being attuned saw new possibilities for action. For example, in being attuned to both the content and contexts of their research, a hermeneutic approach can enable researchers to pay specific attention to issues that are not often attended to, such as the importance of humanities in professional education, how patients are treated as objects in research, system change, or clinical care. A hermeneutic approach sets up a receptivity to evidence and its implementation that can help address the underpinnings of implementation. The six studies show that a fairly direct line can be drawn from a hermeneutic understanding to implementation, to informing a larger project of implementation, or to decision-making. By questioning and articulating assumptions along with bringing to light what is taken for granted or overlooked, it is possible to gain new lines of sight, bring more varied understandings to bear, and imagine and create new solutions.

### Implementing through actions: processes of fusing horizons

Actions are an inextricable part of a hermeneutic approach to implementing, in focusing on ‘*how*’ implementing happens and can happen, rather than on the ‘*what*’ that is to be done. Although the outcome of a hermeneutic approach is frequently conceptual knowledge, gaining that knowledge or awareness comes through the interplay of action and reflection.

In a hermeneutic approach to implementing, reaching a “*fusion of horizons*” [9] is an ongoing process. The horizons are one’s pre-judgments or understandings of a situation, and the fusion happens through dialogue—with others, or with a text. As shown in the six studies, a fusing of horizons extends understanding, creates new knowledge, and leads to action over time through dialogue among those who are leading and those who are participating in implementation. Dialogue is key to exploring and characterizing the problem and possibilities, and especially for knowing that the evidence or other implementation object is right for that context. Common understandings amongst a team implementing a program, service, or practice can be deepened or extended through joint exploration, and concurrent reflection. This process happens in a reflective space, in a particular context, through repeated processes of action and reflection over time. Through developing common understandings and contextually appropriate actions, it is possible to co-create and co-implement processes that result in successful implementing.

### Principles and potential actions in a hermeneutic approach to implementing

On the basis of our findings and interpretations, we have identified recurrent principles of a hermeneutic approach to implementing a program, service, or practice. They are outlined in Table 5. We propose potential implementation actions that flow from and are aligned with the principles. Many of the actions are noted in one or more of the six studies.

**Table 5:** Hermeneutic principles and potential implementation actions

Hermeneutic principle	Potential implementation actions
Acknowledgement of the central concerns of power, communication, and common actions which are active within an issue or problem	• Explore why evidence or ‘object’ needs to be implemented in this particular situation at this time • Examine dimensions of the problem or issue that require the implementation of evidence • Acknowledge power and communication concerns within the issue or problem
Promotion of new ways of seeing situations and creating new common understandings throughout the implementation process	• Introduce theories and other perspectives in ways that extend reflection, understanding, and support consistent action • Avoid uncritically implementing pre-determined strategies or templates from theories or frameworks
Collective action that empowers and shows humility and respect	• Include all relevant persons or stakeholders in the dialogue, planning, and process of implementing • Build and sustain relationships in ways that will foster trust, dialogue, and reflection • Listen and be open to difference and alternative interpretations • Develop respectful ways of engaging and making decisions
Actions that surface tensions and work with context in ways that illuminate possibilities for implementation	• Foster dialogue to explore different positions and views • Explore how initiatives have been, or are currently, achieved in this group, setting, or practice • Explore how usual ways of achieving goals may be connected to the proposed service, program or practice • Seek to understand how people and organizational processes can be engaged in implementation actions • Explore differences in understanding and approaches along with opportunities for action
Processes that address context and the interplay of context with the program, service, or practice being implemented	• Consider how to understand and work with the messiness of the health care/patient interface • Identify actions that embody those understandings • Consider how different understandings of the context can be helpful in adapting what is to be implemented and how to go about implementing • Implement in ways that are attuned to local contexts and their realities • Allow for appropriate flexibility, reflexiveness, and adaptation
Actions that foster engagement in dialogue and listening, action and reflection throughout the implementation journey	• Create and use space for reflection • Engage in respectful, reciprocal dialogue that generates new understandings • Adjust implementation approaches and their pacing and timing as understandings develop over time

The principles and actions are not intended to be a ‘what to do’ checklist or tools for a toolbox. Rather they articulate prompts for thoughtful, ongoing attention to the human face of implementing—to the experiences, needs, and expectations of those who lead and participate in implementing change. Taking on a hermeneutic approach is not simply about acquiring and using new knowledge or skills, but about a stance of openness, or disposition towards new learning that is based on an awareness that experience is never completed. It is shown through “the acquisition of a practiced receptiveness and courtesy toward what is strange, unexpected, and that which lies beyond our most immediate cultural horizon” ([63], p. 66). Taking a hermeneutic approach to implementing is not clear-cut. It is muddy and sometimes not easy to do. Nevertheless, it has great potential to address aspects that are critical to successful implementation that other, more direct and linear approaches are unable to address.

A hermeneutic approach to implementing is not about an external person or team implementing change within a program, service, or practice. It is more about involving leaders and participants in co-creation and co-implementation so that the evidence that is implemented, the knowledge that is extended, as well as the implementation processes undertaken may be shaped in ways that fit the context and the people in it. A hermeneutic approach to implementing demands humility and responsiveness. It engages those involved in ways that enable them to create their own solutions that might not otherwise be imagined. In this way, a hermeneutic approach to implementing has echoes within the tenets of integrated knowledge translation and other similar approaches to research co-production [59].

### Limitations

We followed the guidelines for a scoping review [7] as there was very little literature on the intersection between hermeneutics and implementation to guide the study or understand the field. To maintain conceptual consistency with hermeneutics, we conducted the review in ways that drew on central hermeneutic principles. We kept the search very broad in an attempt to be open to different understandings of hermeneutics and implementing. We may have missed, however, those studies published in theses, dissertations, and the grey literature. We acknowledge the potential charge of subjective bias [7]. We fully embraced and worked with difference and addressed the various perspectives that come with interpretation. The team was inclusive, incorporating members of various disciplines, backgrounds, and perspectives: researchers, a librarian, health service decision-makers and a patient. As almost all members of the research team were actively engaged in reviewing the articles and selecting those included, there was broader involvement than usual in the selection, abstraction, and analysis steps for a scoping review. The potential threat to reliability due to this greater involvement was offset by drawing on all members’ theoretical and practical expertise in analysis, hermeneutics, and implementation. The multi-stage data abstraction process as described may be reproduced by others. At each stage, we considered what counted as hermeneutics and implementation, which required fully engaged dialogue and careful discernment. This process allowed us to come to a common understanding of how a hermeneutic approach to implementing was reported in the literature. At the same time, we were able to refine and clearly articulate our understandings of hermeneutics and implementing that informed our decision-making [39].

There is also little literature about involving patients in knowledge translation and implementation science methodological research [2, 64]. The GRIPP 2 Short Checklist [65] is appended in Additional file 1. As the patient was a full member of the research team since its formation, we have not separately described methods for PPI in the study. The patient’s openness and skill in communication supported receptivity and courtesy within the team. It led other team members to be confident that they could be open about their own gaps in knowledge. As a result differences were not hidden and consensus could be reached with confidence. The experience may well have differed, with a resultant effect on the study, with a different patient and other research team members.

## Conclusions

This scoping review sought to answer the question: what constitutes a hermeneutic approach to the process of implementing health programs, services, or practices? Even though many individual studies incorporate hermeneutics within the research method or draw on hermeneutics to examine patients’ and providers’ experiences of care with recommendations for implementation to follow, only six studies presented a hermeneutic approach to implementing. The great variation in geography, time, philosophical approach, and focus demonstrates the limited attention in the literature given to linking hermeneutics to the process of implementing in the context of health care.

A hermeneutic approach, which focusses on processes and how they happen over time at the actual point of implementing, draws attention to the human endeavor that is implementing, including its inherent need for flexibility in process and recognition of how meanings change over time. This attention is critical if more implementation endeavors are to achieve their goals.

Advances in implementation and implementation research can happen through further studies of hermeneutically grounded implementation and how they achieve their outcomes. Such studies have the potential to extend and refine the hermeneutic approach principles and actions and to provide new insights into effective implementation. A hermeneutic approach to implementing has a philosophical base that may be unfamiliar and challenging for many engaged in implementation science. A productive approach may be found in hermeneutics itself by embracing the differences and fostering dialogue between implementation science researchers, hermeneutic researchers, healthcare practitioners, service providers, and patients/the public. Through a synergistic fusion of horizons among these players, it may be possible to create new ways of approaching implementation and implementation research.

## Supplementary Information

**Additional file 1.** GRIPP2 Short Form.

## Data Availability

The data used to support the findings of this study are available from the corresponding author upon request.

## Abbreviations

ab: Abstract
BC: British Columbia
ED: Emergency Department
KT-IS: Knowledge Translation-Implementation Science
POR: Patient-Oriented Research
SUPPORT: Support for People and Patient Oriented Research and Trials
ti: Title
