# Supplementary material for: Exploring the intersection of hermeneutics and implementation: a scoping review
Source: Syst Rev. 2023 Mar 2;12:30. doi: 10.1186/s13643-023-02176-7 (PMC9979573; doi:10.1186/s13643-023-02176-7)
Supplement: Supplementary file 1 — Additional file 1. GRIPP2 Short Form. [file 13643_2023_2176_MOESM1_ESM.docx]

**Additional File 1: GRIPP2 Short Form**

| Section and topic | Item | Reported on page No |
| --- | --- | --- |
| 1: Aim | Report the aim of PPI in the study | Page 3 |
| 2: Methods | Provide a clear description of the methods used for PPI in the study | n/a The patient was a full member of the research team from the beginning of the project. No specific methods for PPI were used.  See p. 7  See reference 64 for further information. |
| 3: Study results | Outcomes—Report the results of PPI in the study, including both positive and negative outcomes | No specific results – as the patient was a full member of the team, and as such contributed to all results. |
| 4: Discussion and conclusions | Outcomes—Comment on the extent to which PPI influenced the study overall. Describe positive and negative effects | Pages 31-32 |
| 5: Reflections/critical perspective | Comment critically on the study, reflecting on the things that went well and those that did not, so others can learn from this experience | Not directly addressed in this paper. Reference made to the self-study of the Knowledge Mobilization-Implementation Science Methods Cluster projects (reference 64 MacLeod et al., 2022) |

PPI=patient and public involvement

From: Staniszewska S, Brett J, Simera I, Seers K, Mockford C, Goodlad S, et al. GRIPP2 reporting checklists: tools to improve reporting of patient and public involvement in research. Res Involv Engagem. 2017;3:13. <https://doi.org/10.1186/s40900-017-0062-2>
